# Supplementary material for: Temporal analysis of academic performance in higher education before, during and after COVID-19 confinement using artificial intelligence
Source: PLoS One. 2023 Feb 27;18(2):e0282306. doi: 10.1371/journal.pone.0282306 (PMC9970089; doi:10.1371/journal.pone.0282306)
Supplement: Attachment — Submitted filename: ResponseToReviewers.docx [file pone.0282306.s003.docx]

**Additional Editor Comments:**
Notation is line number + "quote" from your paper (if any), then comment

Overall evaluation
The paper has merit in addressing questions of current interest and using an interesting approach (these are the reasons of the 2nd reviewers more positive judgement). However, is suffers from the following major weaknesses:
– Insufficient background and link to existing research on educational background: very little on learning strategies, nothing on procrastination. A similar statement hold for methodology.

The present version of the manuscript includes a detailed introduction and background including the concepts suggested by the reviewer. Moreover, we have also explicitly explained the contribution of our article in the present scenario.

– Insufficient background on methodology, related to a writing style with a focus on code instead of meaning.

Methodology has changed almost completely and now we have a methodology based on education and formal procedures in the main text of the article. The parts of the methodology related to numerical calculations and computation has been moved to appendix.

– disconnect of research questions and of (i) methods (e.g. no significance test for the purported differences); (ii) results (most notably no proper presentation nor explanation of the purported profiles); and thus of (iii) methods/results and conclusions, which to a large part cannot be connected to the data and their analysis.
Further details regarding these (and a few other points) can be found below.

We mostly agree with the editor in the sense that some points were not clear enough in the article. However, since further details will be found below, we will answer to those points there in order to not repeat the same arguments in two places.

For these weaknesses (stated btw by both reviewers) I agree with reviewer 1 to reject the paper. I am sorry to have no better decision at this time, but in view of the interest in principle of your work I encourage you to consider a better embedding and analysis of your data and to draft a new manuscript on this basis. Note, however, that this would have to be an entirely new article.

We really appreciate this comment and that is the reason we are resending this new version of the manuscript taking special care to all the points raised by the editor and reviewers.

87 1. "Is the temporal representation relevant in the students’ profiles?"

unclear (in particular the word “representation”): Do you mean “Are there different time courses of engagement”?

This research question is a technical one in the sense of answering if the classification methods used in the manuscript are working well in the task of classifying students based on their time management.

We agree with the reviewers in changing the style of the manuscript and avoid many computational details, which can be better located in an appendix. For that reason, we have removed this research question since it will be automatically answered by improving the methodology and results sections.

145 "2.2 Data cleaning"

It is confusing to list your variables (“attributes”) the first time under “data cleaning”, and using their code name instead of meaningful terms is obscure and not helpful for the reader to follow your paper. This is a (minor) example of a writing style with a focus on code instead of meaning, see 185 below.

We have included a subsection devoted only to explain the parameters. In this new subsection, we do not use code names. The sections with these technical details are moved now to an appendix as reviewer suggested.

169 "In unsupervised learning, several algorithms can be applied such as k-means, hierarchical clustering and Density-Based Spatial Clustering of Applications with Noise (DBScan)"

First, without proper references, this is just “term dropping”.
Second, and more importantly, unsupervised learning is an educational approach, while these algorithms are clustering methods for data analysis; the connection you have in mind stays completely unclear.
174
elbow method: references to provide the necessary background are missing (the “elbow” method seems to be similar to the “scree” method)
PCA: the rationale of combining it with clustering has to be explained, including again references to provide the necessary background

Unsupervised learning is an educational approach indeed, but it is also the name used for a numerical methodology used in classification algorithms. For avoiding confusion here, we have rewritten the sentence and explicitly explain that we are talking about the algorithms all the time. We have also included references where this numerical methods are explained.

185 "2.4 Regression"
Here, the focus on code – instead of meaning and statistical method – is most salient and a major weakness of the paper. The reader is not interested to see code lines (except perhaps in the appendix), but the statistical method behind it (including pertinent background literature), and how well your data fulfill the prerequisites of its application.

As we have mentioned already, we have changed the focus of the manuscript and we have moved many technical details to the appendix. We have also added indeed more content related to the discussion and data analysis in the terms the reviewers and editor pointed out. As the editor said, the article is now pretty much a new article in the sense of the indexing and structure, but keeping all the contents that made it interesting enough for publication.

Fig 2: Insert supposed to show different user profiles, but completely incomprehensible: how does one recognize e.g. “last-minute” students there? (even the axis captions Comp1 etc. are not explained). This is a major weakness of the paper in its present form (note that while the differentiation among last-minute students etc. is a central point according to the abstract, the term does not appear later on in the text).

Since we have changed the names and interpretation of results in the main body of the article, figures have been changed accordingly. Now, technical details have been removed from the figures (or more technical figures have been moved to the appendix).

231 "In Table 2 we can see that there are three types of profiles of students"
Table 2. Description of the three clusters. Mean is used for the dataset attributes for each cluster

Central statement of your paper, but link to your data is very unclear: One cannot “see” from Table 2, that there are three clusters, it presents some data of these. Then, Table 2 is incomprehensible: What does the caption mean? Do you want to say: Means are presented for each cluster? What are these values ? (is “mean” = “mean mark”; “size” = sample size? and so on)

Indeed, tables have now new names based on the less technical names included now in the main body of the article.

255 (and similar below): "Best parameters of the regression prediction have been 'MLPRegressor activation': 'relu', 'MLPRegressor hidden layer sizes': (4, 4), 'MLPRegressor max iter': 100, 'MLPRegressor solver': 'lbfgs'"

Incomprehensible and uninformative “code jargon”; a reader is interested in the significance and outcomes of your variables, but not their names in the code.

We have revised all the technical details and moved to appendix some contents. To make it easier to understand, we have also included references related to numerical approximations and simulations.

Table 3 is incomprehensible
– what are these values?
– "Malpractices being strict (Error >2)": what does that mean?

This table has been removed and explained in a paragraph. Error > 2 meant that the difference between marks and predicted marks is > 2, which is also explained now in the article.

311 "Does the profile of students vary before, during, and after the COVID-19 pandemic?"

From which data does this follow? Did you run a significance analysis?

Significance analysis were done, but not explicitly shown in the article. This mystake has been corrected in the present version.

**5. Review Comments to the Author**

**Reviewer #1:** Dear Editors, dear authors, while the topic of predicting student success is highly relevant and very interesting, I cannot favor publication of this work in the current form. My main criticism refers to weak reporting of the methodology (that needs strong revision), see below. Moreover, the reporting of the results must also be improved significantly to better guide the reader and to establish a better connection to the RQs.M any statements need to be clarified or supported with additional data. Overall, I am not convinced about the impact of this study or what a reader would learn from it. The most interesting part, in my opinion, is the occurrence of different student groups within the data obtained by cluster analysis /those who continuesly work and pass the exam against those who work hard at the end of the term). However, this is well known (both from experience as a lecturer and from literature). Furthermore, I do not see how this is related to what followed, i.e. a prediction analysis based on different data sets. So despite being a study on a timely issue, I cannot recommend publication. Below you find questions and remarks regarding the text that the authors can use for improving their work.

We really appreciate reviewer opinion, and we agree the paper is not well-presented since it included many technical computational technical details mixed with conclusions and analysis related to education. This is a very difficult equilibrium that mut be carefully included in all interdisciplinary articles. We apologize we did not do it correctly.

It is true that it is well-known that students who worked continuously have a higher chance of success. In that sense, we agree that it would be enough by itself to justify the publication of an article. However, in this article we analyze students’ behaviour before, during and after the COVID19 pandemic. By analyzing these three periods, we have stablished that students worked and managed their time differently. In our opinion, this is a very interesting and new analysis that can be very interesting for readers. In this new version of the manuscript, we make clearer this point and increase the analysis and discussion about what we found related to time management in these three periods and the possible explanations of the differences based on the different circumstances in the three periods. We have also included an extended analysis of the possible solutions and future ways for solving these issues.

RQs on page 3: needs revision: 1) I do not understand at all. 2) Not only does it vary, but to which extent? 3) This is a yes-or no question. Please define / operationalize what is meant with wrong learning strategies 4) do you want to compare data sets? Maybe introduce them first, then refer to them. Again, I suggest avoiding a yes-or no- RQ.

We have rewritten the research questions in order to avoid yes/no questions and to make them easier to understand. First research question was a technical one that can be answered by analyzing the numerical results (details are moved to an appendix in the present version). “to which extent” is something that we have included in all the questions that can be analyzed in that way. Moreover, we have included a couple of questions now related to the interpretation of the results and ideas to improve students’ performance in the future.

We have also changed the paragraph previous to the research questions in order to define all the concepts included in the questions.

Page 3: “Students have been informed by the lecturers that some information about their
activity could be anonymously collected for statistical purposes.”
Did students give their (written) consent to take part in the study. Your statement seems a little but vague to me concerning data protection issues.

The statement has been modified to make it clearer. It has been also moved to a more adequate section.

Main criticism: Was the final exam the same every year? If not, maybe easier exams explain better scores, independent of the year and the COVID circumstances. Please clarify.
Page 3: Methodological issues: Please provide a study plan. It was hard for me figuring out when the tests were applied, and as far as I understood (see bullet points in page 5), students could take the test more than one time before the examination. Please clarify

Please also provide any data you have collected about the participants here (gender, age, …)

We agree with the reviewer we did not give an adequate methodology section since all these details are not correctly explained in the article. We focused the methodology in numerical methodologies instead of the educational protocols and data acquisition. Now, the numerical part is in an appendix and the methodology related to education is in the main article. All the details asked by the reviewer in these points are extendedly explain in the methodology section now.

Page 4: please show an item that you used for assessing students’ scores. I would also suggest giving the complete test as a supplemental material

We have included now a couple of questions as an example. We have included level 1 and level 4 questions in order to show questions of the easiest and more difficult levels.

The complete test is not easy to show because it is composed by 50 core questions with parameters that change every time a student uses the application. This fact implies also that correct answers always change. Moreover, it is part of the official evaluation process in the university and some legal issues may apply if we show it completely.

Page 5: Section 2.3 comes too soon for a reader. Please motivate why you would use this unsupervised method. What is the main reason for applying it here, regarding your RQs.

In the present version of the paper, we have moved all the technical details to the appendix, so now we have a full new methodology section before section 2.3. Moreover, we have included a paragraph which explains why we use unsupervised learning methods. The main reason is to avoid any potential prejudice by the researchers which could appear if we use supervised learning algorithms, where data should be linked to any additional magnitude such as their final marks.

Page 6: The regression details could go in an appendix. Please use words to explain what was done

Regression details are in an appendix now. We have explained how it was done.

Page 7: Can you provide numbers about the distance between the clusters? A dendrogram would help here identifying how far the clusters are separated.
I would reconsider the label of the groups (do not refer to them as “black”, “grey”, etc.)

We have included a dendrogram now and replace the labels in colors by more understandable names.

Page 8: “It can be appreciated that there is a high correlation between month1 and
month2, month2 and month3, and month3 and mark, and that some correlation values
are negative (correlations between month1 and month3count and month2 and
month3count).“
What does this tell us? I would also reconsider the labels of these variables.

We have changed the name of the labels and describe the effects underneath the correlations found in the numerical results.

“This could potentially be caused by malpractices such as copying.”
What do you mean with copying? Copying when taking the CAT, or the final exam? How is this even possible? Otherwise, please clarify.
How have the thresholds been established?

All these details are explained now in the article. In general, copying can happen both in self-evaluation and final exams since students are using adaptive test with a final numerical mark. The thresholds were a couple of examples but, at the end of the day, the limit is fixed by teachers based on their own experience, as we explain now in the present version of the article.

Page 10: We can see that the number of potential malpractices is higher when having less data.”
Do you mean that it is an statistical artefact, and not a “real” effect? Again, what malpractices are you referring to?

We have removed this sentence because it is true it does not have any sense. Potential malpractices can be only detected at the end of the final exam, so it does not make any sense to try to predict them with less data than the whole dataset. Potential malpractices are related to copying of getting information to answer the exam by forbidden sources.

Page 11: “In Fig. 7 a summary of MAE results is shown. We could see that the lowest MAE is
the prediction of 2020/2021 (0.80), followed by all courses (0.88), all courses one month
before (0.92), prediction of 2018/2019 (0.93), and finally the prediction of 2019/2020
(1.01).“
How can a prediction be >1.00?
How can you tell whether the difference of the prediction models is statistically significant? (is .88 really worse than .92). At a first glance, they all look pretty well.

The reviewer is right in pointing out this question since it was not clear in the article. MAE is not giving the percentage of good predictions. It is a numerical parameter that implies a better prediction when it is low. Now, all the details are explained in the article and we have improve the sentences to avoid confusion.

Page 12: no need to repeat the RQs.

It is true we do not need to repeat RQs here since they are already proposed in the article. However, we think it is easier for the readers to have them written again in the discussion section just in case they want to check them.

Page 13: “These results are in agreement with [41], where students who
work continuously have a smaller chance of failure. It is worth noting that students who
work mainly at the end of the term usually succeeded in their objectives. However, the
risk of failure is much bigger for these“
Why is the risk of failure bigger? Where does your data support this, please clarify.

The present version of the manuscript includes a more detailed version of the results and discussion where these details are discussed in detail.

“In terms of data analysis, it is a problem
since it is difficult to analyze data removing the last days or weeks because we lose the
most important part of the information”
This statement is somewhat obvious, I do not get the point here.

It is true it is obvious but we need to define what (even obvious) factors are contributing to the problems we found when trying to predict with less data. It is obvious that less data will give worse predictions. However, it is not obvious which part of the data is the most important, which is why we explain it. In the present version of the manuscript, we have improved the discussion of this point to make it clearer.

“This information is
very interesting since it demonstrates that some good habits lasted one more year at
least.“
What do you mean with good habits? Without asking students about their attitude or work ethic, I think it is very hard to make such conclusions. Maybe the students were just more likely to take the CAT since they got used to online teaching? And they performed better in that period of time just because of that?

We have an extended and improved version of the discussion which explain all these details. Moreover, the reasons why students use CAT often are also explained in the methodology.

**Reviewer #2:** I have reviewed the article “Temporal analysis of academic performance in higher education before, during and after COVID-19 confinement using artificial intelligence”, which presents a large-scale regression study that was conducted with about 400 university students to investigate different profiles of students undertaking online learning. This study found three types of students, with the first type being continuous students, the second type being last-minute students, and the third type being low performing students. I was quite intrigued by the article’s title and abstract and I enjoyed reading this manuscript. Below, I list a few points that I suggest need to be addressed in a revised version.

We really appreciate reviewer comments and his/her good opinion about our manuscript.

1) The introduction sections of the article can be strengthened. Specifically, the last two paragraphs of the first section don’t seem to fit the introduction and would rather support the “Purpose” section. Furthermore, the “Background” section gives a somewhat extensive overview of contemporary research related to the effects of COVID-19 in education. However, most of these previous studies are only mentioned without giving any details about their contents and claims and how they relate to the study in questions. I suggest to revisit the introduction sections and strengthen this important part of the manuscript to allow the readers to properly understand the reasoning behind the article.

We have revised and rewritten both sections following reviewers’ suggestions, including now a better link between the background and the contribution of our paper.

2) The Methods section is missing several important details. For instance, the title of the article does state that it is related to higher education. However, only little information is shared about the setting of the study. Indeed, unless I missed it, I don’t think the article states the age group or the year the students are in. I would suggest to add more information about the study setting – especially in light of an international readership that might not be familiar with the Spanish higher education system.

We have completely revised the methodology section including the details pointed out by the reviewer. Moreover, we have moved some technical details to an appendix for clarity.

3) On page 6, MAE is introduced and its selection is motivated by stating that “it is one of the used metrics used in the literature”. Please add references supporting this claim.

We have added references and explain the method a little bit better in order to make it easier to understand to the readers.

4) I don’t mind the use of grey, light grey and black to distinguish the three types of students in figure 2. However, both in the text and in table 2, I would strongly suggest to rename them with clear and unambiguous labels to avoid potential confusion.

We completely agree with the reviewer and we have changed the three color by names with significance. Now, figures and tables are clearer to read and understand.

5) I have to admit, I’m still pondering the author’s claim that their data shows that students study continuously or only at the end. After all, this is a rather bold inference from the data that relies on test scores. For instance, I’m wondering if the authors considered that type 2 students have been studying continuously as well, but took longer to successfully complete the assignments? I would be curious to read the authors’ evaluation and maybe a cautionary note within the article regarding their claim.

It is true that, with the version of the manuscript that we presented before, these points are not clear at all. In the present version of the manuscript, we have included a full subsection in the methodology to make it clear. The methodology and teaching protocols in the subject under study are a very good guarantee to think that students use the application in most of their autonomous learning. Moreover, all the tasks proposed to the students can be finished in less than two hours so we cannot consider the fact that it takes a longer time (We also measure the time they are connected from the beginning to the end of their tasks). A longer and more detailed version of this explanation is included in the article.

6) Personally, I’m not a big fan of research questions that can be answered with yes/no. The authors do a good job answering the first three questions with sufficient detail, but the discussion section for research question 4 clearly fell short, as indicated by the rather tentative statement in brackets at the end of the section. I would suggest strengthening this important part of the manuscript by adequately discussing the findings of the study and putting it in a broader context with existing research from the literature. (This comment ties back to my first comment about the introduction section.)

We have modified all the research questions and now all of them are not “Yes/No” questions. We have increased the discussion in all of the new research questions, including indeed contents related to the old fourth research question.

7)Last, I would be curious to learn the authors’ suggestion how teachers can “correct wrong learning strategies”, as mentioned in the conclusion section. I think answering this question, even if only tentatively, could make a significant contribution to our field.

We have added a RQ related directly to this point.

Overall, I am in favour of this manuscript. Occasionally, there are grammar issues and spelling mistakes that need to be corrected. I suggest minor revisions before its acceptance.

We appreciate this opinion of our article and we have made a considerable effort to correct it in the way suggested by the reviewer.
